# Synthesis, structure and orange-light emission of a zero-dimensional anti­mony(III) halide with the 4,4′-(ethane-1,2-di­yl)di­pyri­din-1-ium cation

**DOI:** 10.1107/S2053229626006935

**Published:** 2026-07-20

**Authors:** Ting Li, Yan-Hong Li, Jia-Yi Han, Yi-Tong Wang, Min-Le Han

**Affiliations:** a College of Chemistry and Chemical Engineering Luoyang Normal University,Luoyang 471934 People’s Republic of China; b Department of Enviromental Scinece and Engineering North China Electric Power University (Baoding) Baoding 071003 People’s Republic of China

**Keywords:** crystal structure, 1,2-bis­(pyridin-4-ium)ethane, 4,4′-(ethane-1,2-di­yl)di­pyri­din-1-ium, anti­mony hydrid halide, orange emission, lead-free halide, IOMH

## Abstract

Exploring orange-light-emitting lead-free halides with high photolu­mi­nes­cence quantum yields (PLQYs) is a significant challenge in lu­mi­nes­cent materials research. Herein, a zero-dimensional organic–inorganic hybrid halide, namely, 4,4′-(ethane-1,2-di­yl)di­pyri­din-1-ium penta­chlorido­anti­monate(III), (C_12_H_14_N_2_)[SbCl_5_] or [H_2_bpa][SbCl_5_], where bpa is 1,2-bis­(pyridin-4-yl)ethane, was syn­thesized *via* acid solution evaporation. The com­pound crystallizes in the triclinic space group *P*1. The asymmetric unit consists of an [SbCl_5_]^2−^ anion and two half [H_2_bpa]^2+^ cations. The [SbCl_5_]^2−^ anion and [H_2_bpa]^2+^ cations are linked *via* inter­molecular hy­dro­gen bonds and π–π inter­actions to form a three-dimensional supra­molecular architecture. Significantly, this halide exhibits highly efficient orange-light emission centred at 600 nm, with a PLQY of 65.57%.

## Abstract 2

The hybrid halide [H_2_bpa][SbCl_5_] [H_2_bpa is 4,4′-(ethane-1,2-di­yl)di­pyri­din-1-ium] exhibits efficient orange emission centred at 600 nm with a photolu­mi­nes­cence quantum yield of 65.57%.

## Introduction

Recently, zero-dimensional (0D) inorganic–organic metal halides (IOMHs) have shown tremendous potential as lu­mi­nes­cent materials due to their diversified structures and tunable photolu­mi­nes­cence (PL) (Han *et al.*, 2021 ▸; Haque *et al.*, 2025 ▸; Cong *et al.*, 2025 ▸; Zhou *et al.*, 2026 ▸; Toumi *et al.*, 2025 ▸; Zouari *et al.*, 2026 ▸; Zhao *et al.*, 2026 ▸). Generally, 0D IOMHs are com­posed of isolated metal halide clusters or units and bulky organic cations with a richer structure database and assembly framework (Shentseva *et al.*, 2024*a* ▸; Shentseva *et al.*, 2024*b* ▸; Shentseva *et al.*, 2025*a* ▸; Shentseva *et al.*, 2025*b* ▸). In contrast to high-dimensional halides, 0D IOMHs always show strong quantum/dielectric confinement and possess much softer lattices, leading to an increased exciton binding energy and allowing for self-trapped exciton (STEs) emission with a wide broadband and large Stokes-shifted properties (Morad *et al.*, 2019 ▸; Dastidar *et al.*, 2024 ▸; Wang *et al.*, 2024 ▸). Up to now, lower-energy green, yellow, orange and red emissions have been readily realized in 0D halides.

Sb^III^-based metal halides with 5*s*^2^ lone pairs exhibit the unique advantages of long-wavelength emission in 0D emitters. Great efforts have been devoted to exploring the preparation of 0D lead-free Sb^III^-based IOMHs (Zhou *et al.*, 2018 ▸; Huang *et al.*, 2025 ▸; Lv *et al.*, 2025 ▸; Lin *et al.*, 2020 ▸; Vovna *et al.*, 2015 ▸). [Sb*X*_5_]^2−^ units typically exhibit efficient broadband emission due to STEs, which is generated by exciton–lattice inter­actions and structural rearrangement in the excited state (Li *et al.*, 2021 ▸). For instance, Ma and co-workers re­ported an Sb^III^-based 0D hybrid, (C_9_NH_20_)_2_[SbCl_5_], exhibiting a bright-orange emission and a near-unity photolu­mi­nes­cence quantum yield (PLQY) (98 ± 2%) (Zhou *et al.*, 2018 ▸). Du and co-workers have reported a series of 0D hybrid Sb^III^-based 0D hybrids of *A*SbCl_5_ (*A* = [C_3_mmim]^+^, [C_5_mmim]^+^ and [C_5_mim]^+^, where [C_3_mmim]^+^ is 2,3-di­methyl-1-propyl­imid­a­zol­ium, [C_5_mmim]^+^ is 2,3-di­methyl-1-pentyl­imid­a­zol­ium and [C_5_mim]^+^ is 3-methyl-1-pentyl­imid­a­zol­ium) based on discrete [SbCl_5_]^2−^ units exhibiting yellow or orange–yellow emission and a maximum PLQY of 79.5% (Chen *et al.*, 2025 ▸). It is worth noting that many IOMH systems containing [SbCl_5_]^2−^ exhibit high PLQYs (>50%) due to their STEs. This remarkable property has garnered significant attention with respect to efficient solid-state lu­mi­nes­cent materials (Jing *et al.*, 2021 ▸; Wang *et al.*, 2022 ▸).

**Figure 1:**
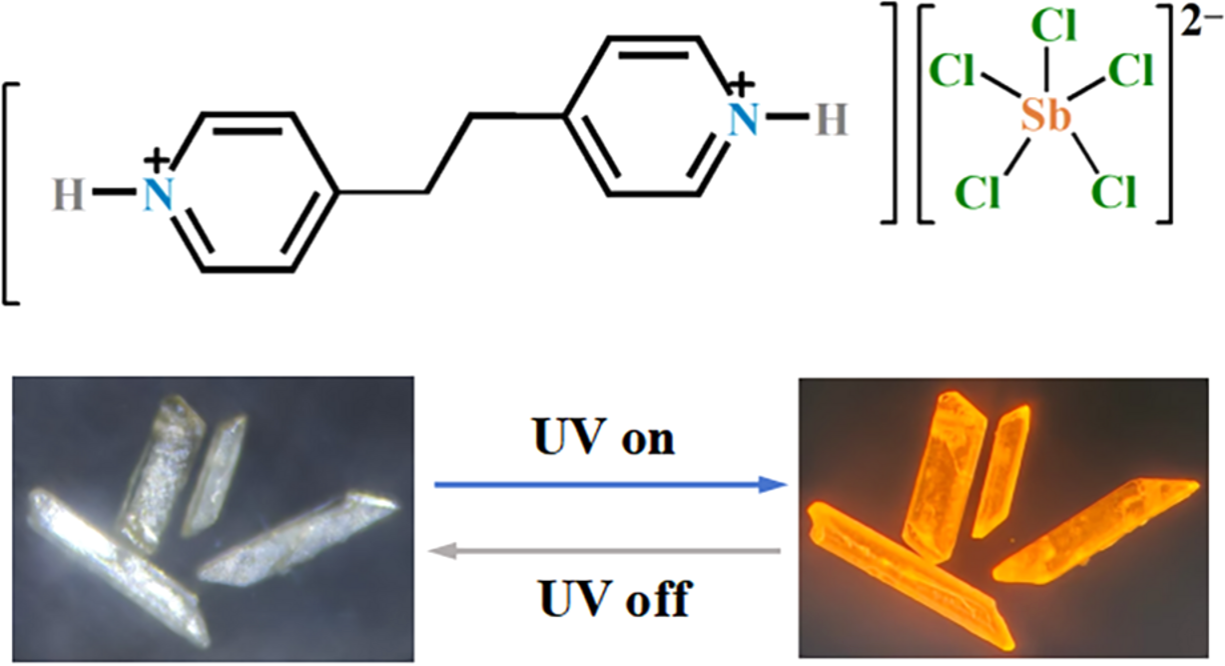
Schematic representation of the structure of [H_2_bpa][SbCl_5_] (top) and crystal photos of the compound under daylight (left bottom) and 365 nm UV light (right bottom).

In the synthetic strategies for organic–inorganic hybrid metal halides, pyridine-based organic mol­ecules, such as bi­pyridine, are commonly used as cation sources. 4,4′-(Ethane-1,2-di­yl)di­pyri­din-1-ium was also adopted as a cation, and two hybrid bis­muth(III) halide crystals have been reported (Adonin *et al.*, 2015*a* ▸; Adonin *et al.*, 2015*b* ▸). However, no hybrid Sb^III^-based halides have been reported using pro­ton­ated 1,2-bis­(pyri­din-4-yl)ethane as the organic cation. In the present study, a new 0D IOMH, [H_2_bpa][SbCl_5_] (Fig. 1 ▸), was prepared using 1,2-bis­(pyridin-4-yl)ethane as a cation source. This halide exhibited an intense orange emission centred at 600 nm, mainly arising from the STEs of the [SbCl]^2−^ unit with a PLQY of 65.57%. This work provides an efficient strategy to achieve high-efficiency orange emission.

## Experimental

### Materials and methods

Anti­mony trichloride (anhydrous, SbCl_3_, 99.9%) and 1,2-bis­(pyridin-4-yl)ethane (bpa, 99.8%) were purchased from Alfa, and hy­dro­gen chloride (HCl, 38%) was purchased from Aladdin. All chemicals are used directly without further purification. Elemental analysis for C, N and H was performed on a Flash2000 organic elemental analyzer. Thermogravimetric analysis was performed on a NETZSCH STA449C microanalyzer heated from 30 to 400 °C in an air atmosphere. Powder X-ray diffraction (PXRD) patterns were taken on a Rigaku D/Max 2500 powder X-ray diffractometer using Cu *K*α radiation (λ = 1.5418 Å) at a voltage of 40 kV and 40 mA. PL spectra, quantum yield and time-resolved decay spectra were recorded on an Edinburgh FLS1000 fluorescence spectrometer with a xenon lamp and an integrated sphere sample chamber.

### Synthesis of [H_2_bpa][SbCl_5_]

A mixture of bpa (0.01 mmol) and SbCl_3_ (0.02 mmol) was dissolved in a solution of HCl (1 ml) and stirred at 353 K for 30 min. Light-yellow block-shaped crystals were obtained by slow evaporation under ambient conditions for 1 d. The crystals were collected, washed with acetone five times and then dried in air (yield 63.5%, based on bpa). Analysis calculated (%) for C_12_H_14_Cl_5_N_2_Sb: C 29.70, H 2.91, N 5.77; found: C 30.24, H 2.94, N 5.83.

### Refinement

Crystal data, data collection and structure refinement details are summarized in Table 1 ▸. H atoms on C and N atoms were located at geometrically calculated positions. The [H_2_bpa]^2+^ cation containing atom N2 is entirely disordered. Rings N2/C7–C11 and N2*A*/C7*A*–C11*A* were disordered over two sites with occupancies of 0.649 (10) and 0.351 (10).

### Density functional theory (DFT) calculations

First-principles calculations were carried out using the projected augmented wave (PAW) method (Kresse *et al.*, 1996 ▸), as implemented in *VASP* (Kresse *et al.*, 1999 ▸; Perdew *et al.*, 1992 ▸). The exchange correlation energy was treated using the Perdew–Burke–Ernzerhof (PBE) exchange-correlation functional in the scheme of generalized gradient approximation (Hohenberg *et al.*, 1964 ▸; Perdew *et al.*, 1996 ▸). The kinetic energy cutoff for all cases was determined to be 520 eV. The convergence thresholds for the electronic calculations and ionic relaxations were chosen as 10^−6^ eV and 0.01 eV Å^−1^, respectively. The standard Monkhorst–Pack *k*-point grids with a density of 0.1 Å^−1^ were used for Brillouin zone sampling. The valence electron configurations applied in this work were treated as Sb 5*s*^2^5*p*^3^, C 2*s*^2^2*p*^2^, Cl 3*s*^2^3*p*^5^, N 2*s*^2^2*p*^3^ and H 1*s*^1^. Owing to atomic disorder, each atom is split into two atoms through symmetry operations, corresponding to the presence of two molecules. For theoretical calculations, one set of these molecules is selected for computation (Wu *et al.*, 2026 ▸; Ogawa *et al.*, 2020 ▸).

## Results and discussion

**Figure 2:**
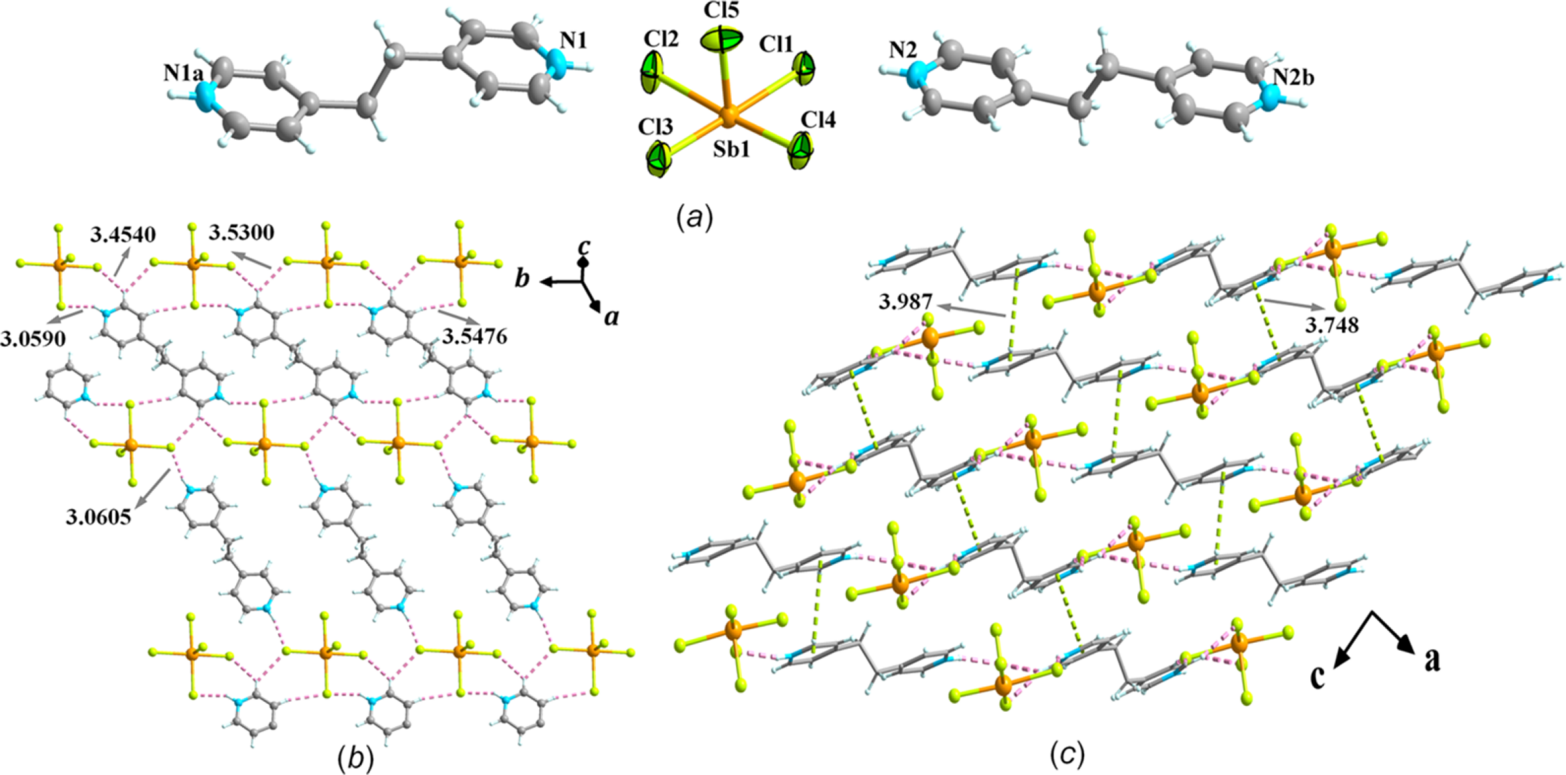
The mol­ecular structure of [H_2_bpa][SbCl_5_], showing (*a*) the pyramidal [SbCl_5_]^2−^ anion and [H_2_bpa]^2+^ cation [symmetry codes: (a) −*x* + 3, −*y*, −*z* + 1; (b) −*x* + 1, −*y* + 1, −*z* + 2], (*b*) the 2D layer and (*c*) the 3D supra­molecular structure.

Single-crystal X-ray diffraction analysis revealed that [H_2_bpa][SbCl_5_] crystallizes in the triclinic space group *P*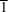. The asymmetric unit consisted of two half [H_2_bpa]^2+^ cations and one [SbCl_5_]^2−^ anion. As shown in Fig. 2 ▸(*a*), each Sb^III^ ion is coordinated to five Cl^−^ anions to form a distorted tetra­gonal [SbCl_5_]^2−^ unit, yielding a zero-dimensional (0D) structure. The Sb—Cl bond lengths range from 2.3610 (6) to 2.7621 (6) Å and the Cl—Sb—Cl angles range from 86.30 (3) to 91.58 (2)°, which are com­parable to literature values (Peng *et al.*, 2023 ▸; Wang *et al.*, 2019 ▸). There exist inter­molecular hy­dro­gen-bonding inter­actions between the [H_2_bpa]^2+^ cations and the [SbCl_5_]^2−^ anions, with N⋯Cl = 3.0605 (19) and 3.0590 (9) Å, and C⋯Cl = 3.4540 (2), 3.5300 (2) and 3.5476 (18) Å. Thus, the [SbCl_5_]^2−^ tetra­gonal pyramidal units connect [H_2_bpa]^2+^ cations *via* inter­molecular hy­dro­gen bonds to form a two-dimensional (2D) layer [Fig. 2 ▸(*b*)]. The two pyridinium rings of one [H_2_bpa]^2+^ cation are arranged parallel to other [H_2_bpa]^2+^ cations in adjacent layers, with inter­planar distances of 3.748 and 3.987 Å [Fig. 2 ▸(*c*)]. Such 2D layers are further stacked into a three-dimensional (3D) supra­molecular architecture. The degree of structural distortion of the [SbCl_5_]^2−^ pyramid was evaluated by calculating the angular distortion parameter (σ^2^) according to the equation of Robinson *et al.* (1971 ▸). The calculated σ^2^ value is 2.91 and such a degree of distortion (Li *et al.*, 2019 ▸; Peng *et al.*, 2023 ▸) suggests the generation of self-trapped excitons (STEs).

**Figure 3:**
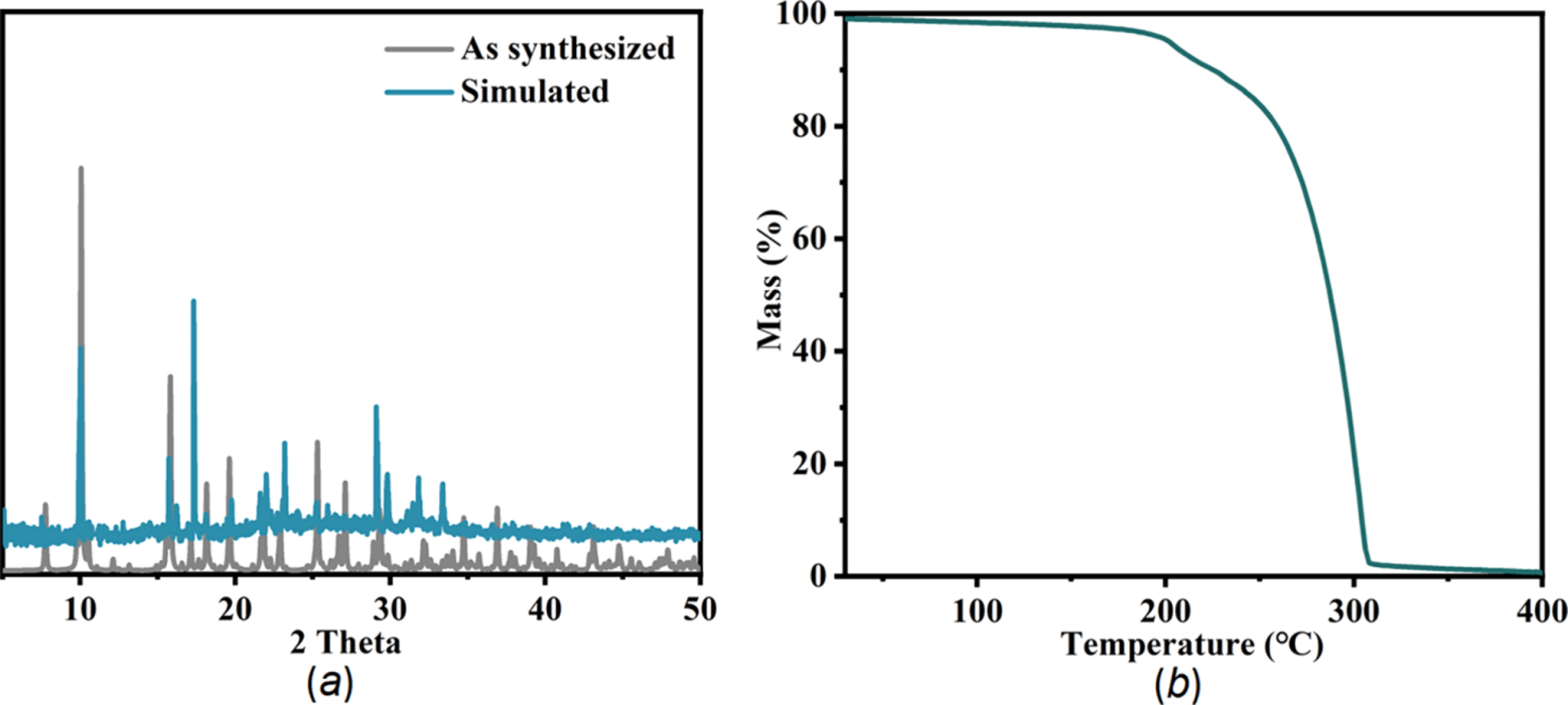
The basic characterization of [H_2_bpa][SbCl_5_], showing (*a*) the experimental and simulated PXRD patterns of [H_2_bpa][SbCl_5_], and (*b*) the thermal analysis of [H_2_bpa][SbCl_5_].

The powder X-ray diffraction (PXRD) pattern of [H_2_bpa][SbCl_5_] matched well with that simulated from the single-crystal structure data, indicating high phase purity [Fig. 3 ▸(*a*)]. The thermogravimetric (TG) curve showed that the [H_2_bpa][SbCl_5_] crystals exhibited excellent thermal stability and begin to decom­pose from approximately 200 °C [Fig. 3 ▸(*b*)].

**Figure 4:**
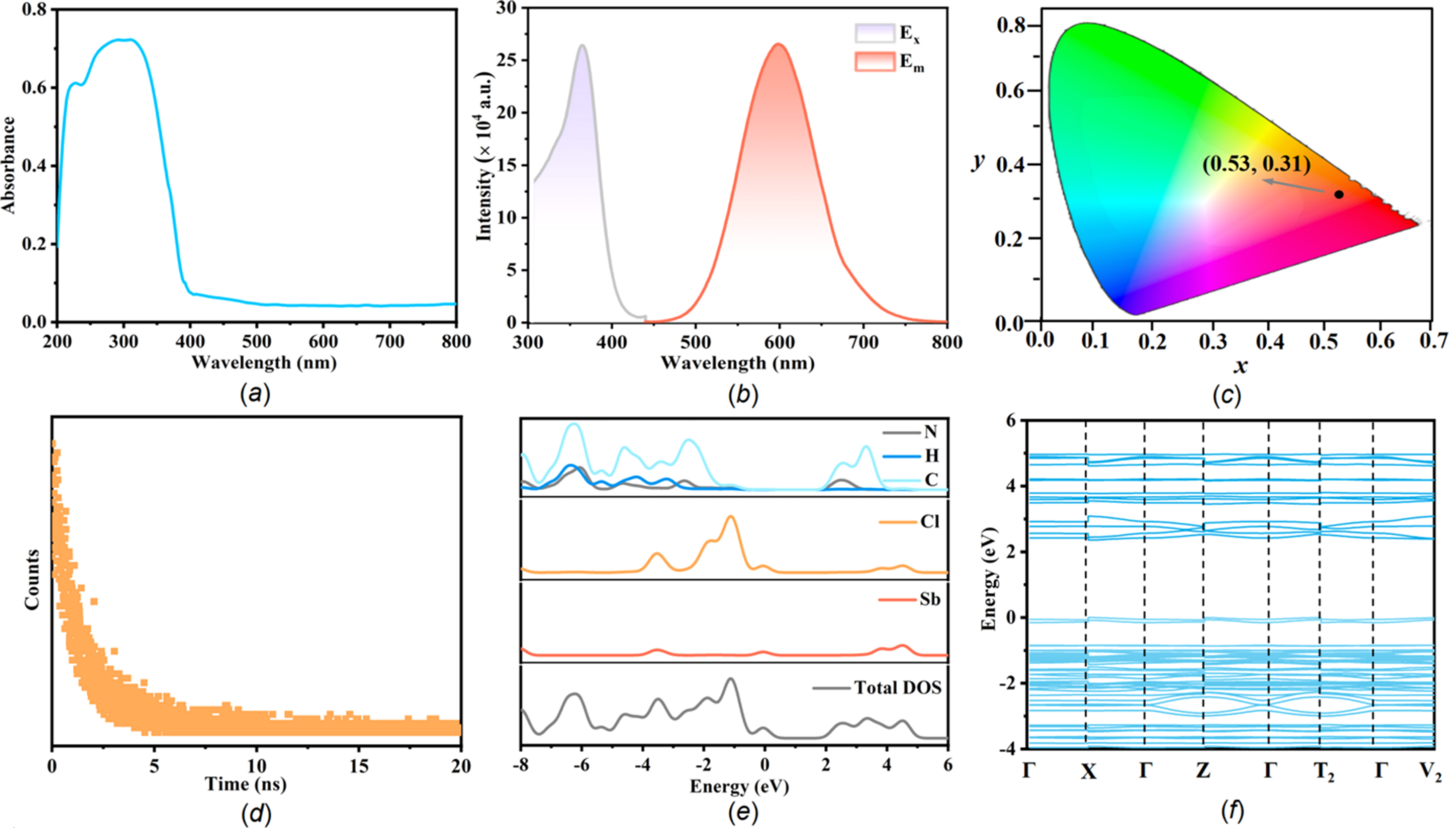
The lu­mi­nes­cence properties and theoretical calculations for [H_2_bpa][SbCl_5_], showing (*a*) the solid-state UV–Vis absorption of [H_2_bpa][SbCl_5_], (*b*) the excitation and emission spectra of the organic mol­ecule of [H_2_bpa][SbCl_5_], (*c*) the CIE chromaticity coordinates and (*d*) the PL decay curves of [H_2_bpa][SbCl_5_]. Theoretical calculations for [H_2_bpa][SbCl_5_], showing (*e*) DOS and (*f*) the calculated band structure.

The photophysical properties of [H_2_bpa][SbCl_5_] were in­vestigated. As shown in Fig. 4 ▸(*a*), the solid-state UV–Vis ab­­sorption spectrum of [H_2_bpa][SbCl_5_] exhibited a band edge at approximately 400 nm [Fig. 4 ▸(*a*)]. To characterize the op­tical properties of [H_2_bpa][SbCl_5_], the steady-state and time-re­sol­ved photolu­mi­nes­cence (PL) spectra were measured. Upon 360 nm excitation, [H_2_bpa][SbCl_5_] exhibited a bright-orange-emitting band centred at 600 nm, with a full-width at half-maximum (FWHM) of 100 nm and a large Stokes shift of 245 nm [Fig. 4 ▸(*b*)]. The Commission Inter­nationale de l’Éclair­age (CIE 1931) chromaticity coordinates were determined as (0.52, 0.46) [Fig. 4 ▸(*c*)]. The PL decay lifetime was 1.52 ns at 600 nm [Fig. 4 ▸(*d*)]. The title com­pound exhibits a PL quantum yield (PLQY) value of 65.57%, which was moderate among this class of com­pounds (Zhang *et al.*, 2021 ▸; Luo *et al.*, 2022 ▸; Peng *et al.*, 2021 ▸; Peng *et al.*, 2022 ▸; Peng *et al.*, 2023 ▸; Li *et al.*, 2019 ▸).

**Figure 5:**
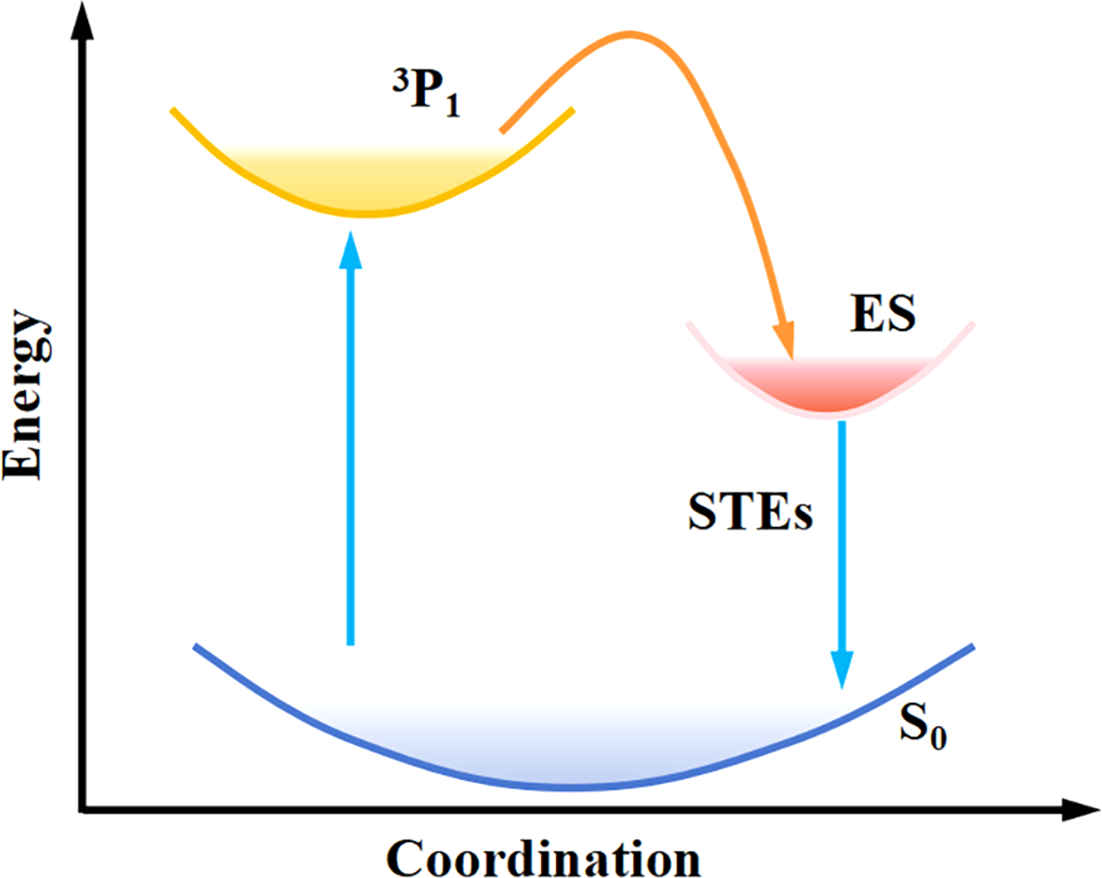
Possible photophysical mechanism for [H_2_bpa][SbCl_5_].

To better understand the lu­mi­nes­cence mechanism of [H_2_bpa][SbCl_5_], density functional theory (DFT) calculations were performed to investigate the band structure and density of states (DOS). The valence band maximum (VBM) of [H_2_bpa][SbCl_5_] was mainly located on the [H_2_bpa]^2+^ cation and Cl atoms, and the conduction band minimum (CBM) was dis­tri­buted over the [SbCl_5_]^2−^ anion [Fig. 4 ▸(*e*)]. The energy band gap between the valence and conduction bands of [H_2_bpa][SbCl_5_] was 2.32 eV [Fig. 4 ▸(*f*)], and such a small value indicates a pronounced quantum confinement effect. Meanwhile, these energy band distributions indicated that there was a spatial charge separation between the [SbCl_5_]^2−^anion and the [H_2_bpa]^2+^ cation, and the charge transfer (CT) may be gen­er­ated from the [SbCl_5_]^2−^anion to the [H_2_bpa]^2+^ cation. Con­sidering the weak blue-light emission from the organic ligand, these results further confirmed that the intense orange emission of [H_2_bpa][SbCl_5_] originated from STEs of the [SbCl_5_]^2−^ nodes (Ren *et al.*, 2024 ▸; Zhou *et al.*, 2024 ▸), and the whole emission contained the contribution of CT from the [SbCl_5_]^2−^ anion to the [H_2_bpa]^2+^ cation (Qi *et al.*, 2022 ▸; An *et al.*, 2024 ▸). The photophysical processes can be summarized as follows: Sb^III^ has a typical 5*s*^2^ electronic configuration, the ground state is ^1^S_0_, the excited states of the 5*s*^1^5*p*^1^ configuration contain a singlet state (^1^P_1_) and triplet states (^3^P_*n*_, *n* = 0, 1 and 2) (Jing *et al.*, 2021 ▸). As shown in Fig. 5 ▸, under excitation at 365 nm, the electrons were excited to the ^3^P_1_ state, followed by inter­system crossing to the emitting state (ES), forming triplet excitons. The transition of triplet ^3^P_1_ to ground state ^1^S_0_ generated the low-energy emission.

## Summary

Using 1,2-bis­(pyridin-4-yl)ethane, an anti­mony-based hybrid halide has been prepared. It exhibited efficient orange emission with a high PLQY of 65.57%. Experimental and theoretical studies indicated that the emission originated from the synergistic contribution of electron transitions in the organic cations and STE states. This work lays the foundation for synthesizing more-efficient orange-emitting hybrid halides.

## Supplementary Material

Crystal structure: contains datablock(s) I, global. DOI: 10.1107/S2053229626006935/son3007sup1.cif

Structure factors: contains datablock(s) I. DOI: 10.1107/S2053229626006935/son3007Isup2.hkl

CCDC reference: 2543274

## Figures and Tables

**Table 1:** Experimental details

Crystal data	
Chemical formula	(C_12_H_14_N_2_)[SbCl_5_]
*M*_r_	485.25
Crystal system, space group	Triclinic, *P* 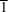
Temperature (K)	279
*a*, *b*, *c* (Å)	7.1856 (3), 8.7084 (4), 15.3066 (5)
α, β, γ (°)	90.921 (1), 91.486 (1), 113.638 (2)
*V* (Å^3^)	876.79 (6)
*Z*	2
Radiation type	Mo *K*α
μ (mm^−1^)	2.33
Crystal size (mm)	0.24 × 0.22 × 0.21

Data collection	
Diffractometer	Bruker APEXII CCD
Absorption correction	Multi-scan (*SADABS*; Bruker, 2016 ▸)
*T*_min_, *T*_max_	0.598, 0.746
No. of measured, independent and observed [*I* > 2σ(*I*)] reflections	42381, 4363, 3975
*R*_int_	0.051
(sin θ/λ)_max_ (Å^−1^)	0.667

Refinement	
*R*[*F*^2^ > 2σ(*F*^2^)], *wR*(*F*^2^), *S*	0.022, 0.052, 1.08
No. of reflections	4363
No. of parameters	245
No. of restraints	225
H-atom treatment	H-atom parameters constrained
Δρ_max_, Δρ_min_ (e Å^−3^)	0.46, −0.54

Computer programs: *APEX2* (Bruker, 2016 ▸), *SAINT* (Bruker, 2016 ▸), *olex2.solve* (Bourhis *et al.*, 2015 ▸), *SHELXL2014* (Sheldrick, 2015 ▸) and *OLEX2* (Dolomanov *et al.*, 2009 ▸).
